# Bioorthogonal tuning of nanosurface opsonisation *via* click coupling of a complement fusion protein inhibitor

**DOI:** 10.1039/d6cc00733c

**Published:** 2026-06-09

**Authors:** Sarah Jacques, Hanmant Gaikwad, Jemma Pillatzke, Seyed Moein Moghimi, Dmitri Simberg

**Affiliations:** a Department of Pharmaceutical Sciences, The Skaggs School of Pharmacy and Pharmaceutical Sciences, University of Colorado Anschutz Medical Campus Aurora CO USA seyed.moghimi@newcastle.ac.uk Dmitri.simberg@cuanschutz.edu; b School of Pharmacy and Pharmaceutical Sciences, Panoz Institute, Trinity College Dublin Dublin 2 Ireland; c School of Pharmacy, Newcastle University Newcastle upon Tyne NE1 7RU UK; d Translational and Clinical Research Institute, Faculty of Health and Medical Sciences, Newcastle University Newcastle upon Tyne NE2 4HH UK

## Abstract

Complement opsonisation contributes to immune clearance of nanopharmaceuticals, but complement activation has some undesirable effects. Herein, we show that tethering the fusion complement receptor 2-complement receptor 1 (CR2-CR1) regulator to cross-linked iron oxide nanoworms *via* a PEG spacer and copper-free click chemistry overcomes complement opsonisation. Furthermore, CR2-CR1-conjugated nanoworms reduce complement opsonisation of unmodified nanoparticles.

## Abstract 2

Tethering of a complement fusion regulator to nanoparticles prevents complement C3 opsonisation and protects bystander unmodified nanoparticles from C3 deposition.

## Graphical abstract

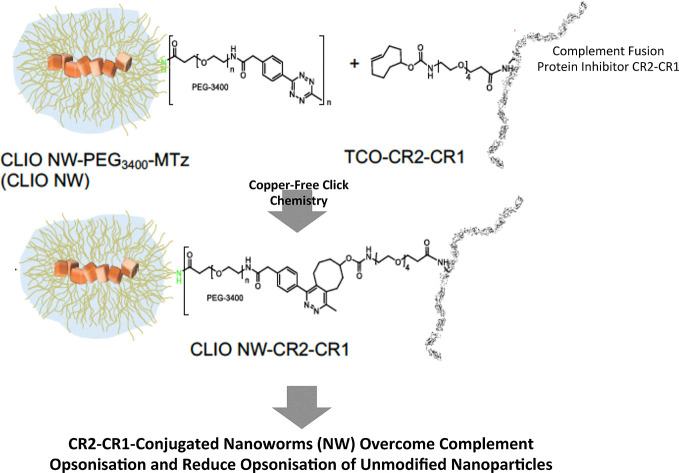

The complement system, a network of more than 50 soluble and membrane-bound proteins, is a critical line of defence against pathogens and particulate matter, including nanopharmaceuticals.^1–3^ Complement is activated through classical, lectin, and alternative pathways, resulting in surface opsonisation of the intruder through sequential cleavage of the third complement protein (C3) (*i.e.*, C3b, iC3b, C3dg and C3d opsonins), the release of anaphylatoxins C3a and C5a, and assembly of the membrane attack complex C5b-9.^1,3^ C3 opsonised nanoparticles are recognised *via* a multitude of complement receptors (CRs) expressed on neutrophils, monocytes, eosinophils, B-cells, dendritic cells and tissue macrophages.^1,3–6^

Nature has developed complement regulators and control proteins strategically positioned on cell surfaces and in the fluid phase. Examples include CD35 (CR1), CD55 (decay-accelerating factor), CD59, CD46, factor H, and C1-inhibitor.^3,7,8^ There has been a long-standing interest in natural complement regulators for treating complement-dependent diseases and protecting post-ischaemic organs.^3,9,10^ Overall, complement therapeutics is a rapidly evolving approach to treating complement-dependent disorders.^9,10^ Examples include orally bioavailable small-molecule inhibitors of the alternative pathway, iptacopan (FABHALTA®) and danicopan (VOYDEYA®), approved for the treatment of paroxysmal nocturnal haemoglobinuria and atypical haemolytic uremic syndrome.^11^ Iptacopan binds to complement factor B and prevents its cleavage into Bb, whereas danicopan binds to complement factor D, which prevents the enzymatic processing of factor B into Bb, thereby preventing the formation of alternative pathway convertases that eventually cleave C3 and C5. Another example is Sutimlimab (ENJAYMO®), a monoclonal antibody approved for the treatment of cold agglutinin disease.^12^ This binds to the zymogen C1s, preventing its activation and the enzymatic conversion of C2 and C4 into the classical C3 convertase C4b2a. The PEGylated derivative of cP40 (pegcetacoplan, EMPAVELI®) is another interesting inhibitor, which exerts its function by binding to the entire pool of C3 and allosterically inhibiting its cleavage by all complement convertases.^13^ Other approved inhibitors include Eculizumab (SOLIRIS®), a monoclonal antibody that blocks C5 and prevents the generation of C5a and C5b-9, and avacopan (TAVNEOS®), a potent antagonist of the C5a receptor, for the treatment of paroxysmal nocturnal haemoglobinuria and vasculitis, respectively.^14,15^ However, most recently, the Food and Drug Administration's Center for Drug Evaluation and Research proposed to withdraw approval of TAVNEOS.^16^

Complement inhibitors are not bioorthogonal, which could lead to an immunocompromised host and increased susceptibility to infections.^17,18^ Sustained complement inhibition increases the risk of intractable, potentially deadly infections that are costly to treat^17,18^ —a concern also highlighted by the United States Food and Drug Administration's iptacopan boxed warning, especially for immunosuppressed patients. Thus, with respect to materials application in medicine and to ameliorate the deleterious effects of biomaterials’- and nanopharmaceutical-induced complement activation, attempts have been made to tether natural complement inhibitors such as CD55, factor H, and factor I to biomaterials and injectable drug carriers *via* chemical modification, lipid anchors, antibodies, or peptidic baits with some degree of success and efficacy in suppressing C3 opsonisation.^19–22^

We recently reported the potent inhibitory effect of short-circulating soluble 120 kDa fusion regulator CR2–CR1, which combines complement receptor 2 (CR2) and CR1, on complement activation in conjunction with nanoparticles.^5,23^ CR2 binds to C3 fragment-opsonised surfaces, whereas CR1 inhibits C3 convertases.^24^ In our studies, we found that surface-targeted CR2-CR1 is much more potent than non-targeted CR1 with nanomolar potency and with multiple nanoparticle types *in vivo*.^23^ CR2-CR1 completely inhibits nanoparticle-mediated complement activation and acute responses (lethargy) in rats after intravenous injection,^23^ without inducing long-lasting complement suppression. These observations present a universal opportunity to develop complement-evading nanoparticles. Herein, we show tethering of CR2-CR1 to 60 nm PEGylated cross-linked dextran iron oxide nanoworms^25,26^*via* methyltetrazine-transcyclooctene not only inhibits C3 opsonisation of the nanosurface, but also CR2-CR1-bound nanoparticles effectively inhibit complement activation by unprotected nanoparticles and PEGylated liposomes.

We used cross-linked dextran iron oxide nanoparticles for modification, since they are a highly versatile platform for targeted *in vivo* imaging and drug delivery.^27^ Crosslinking the dextran coating with epichlorohydrin blocks complement activation in mouse but not in human sera,^25,28^ due to a discrepancy between the mouse and human complement system.^6,25,29^ Cross-linked dextran iron oxide nanoworms were modified with CR2-CR1 (Fig. 1a) as described before for antibody conjugation.^26^ We employed copper-free Diels–Alder addition of strained *trans*-cyclooctene (TCO) and methyltetrazine (MTz). This is a highly versatile reaction due to very fast second-order kinetics and high stability of the resulting bond suitable for biological studies.^30^ First, we prepared a PEGylated intermediate of cross-linked dextran iron oxide nanoworms (SI), CLIO NW-PEG_3400_-Mtz (thereafter CLIO NW), as described previously.^26^ These CLIO NWs had the intensity weighted mean hydrodynamic diameter of 63 nm and zeta potential −2 mV (Fig. 1b). Next, these NWs were conjugated with TCO-modified CR2-CR1 (1–2 TCO/protein as verified by Cy3-MTz) (SI). After the modification, the average number of bound CR2-CR1 was 36/NW as determined in a dot-blot with anti-CR2 antibody (SI). The mean hydrodynamic size of the resultant particles was 78 nm and the zeta potential of +12 mV indicates colloidal stability (Fig. 1b).

**Fig. 1:**
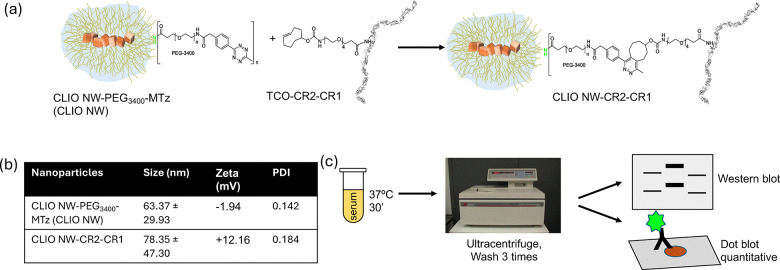
Click coupling procedures of CR2-CR1 to PEGylated cross-linked dextran iron oxide nanoworms (CLIO NW) (a), their hydrodynamic size and electrokinetic potential characteristics (b), and immunological processing procedures in human sera (c). In nanoworm drawings, yellow indicates dextran and brown represents iron oxide nanocrystals. Details of chemical, analytical, and immunological procedures are presented in the supplementary information (SI) file. NWs were colloidally stable for at least 3 months when stored at 4 °C in phosphate-buffered saline and the data presented is based on NWs approximately 1 month after preparation.

To measure total C3 deposition, we used a previously validated quantitative immuno dot-blot as well as western blot^23,31^ (Fig. 1c). In native form, C3 is composed of two polypeptide chains (an α chain of 118 kDa and a β chain of 75.5 kDa) held together by a single disulfide bond.^3^ C3 is activated by the action of C3 convertases on the α chain, releasing a 9 kDa fragment (C3a) from its amino-terminal end.^3^ The remaining portion of the α chain is referred to α′. Nascent C3b (composed of α′ and β chains) covalently binds to reactive surfaces and is rapidly inactivated by factor H in tandem with factor I, releasing a 2 kDa fragment (C3f) into the fluid-phase. Further processing by factor I and other complement regulators eventually releases fragment C3c (22.5 + 39.5 kDa, held by a disulfide bridge), leaving a 45 kDa fragment (C3dg) surface-bound. Further processing of C3dg releases C3g (5 kDa), with C3d (40 kDa) covalently bound to the surface.^3^ Proteolytic fragment of the α′ chain is typically referred to as α′2. Western blotting, therefore, allows determining the C3 activation status *via* deposition of the cleavage fragments of the α′ chain (specifically α′2) on NWs.^23^

Both western blot (Fig. 2a) and dot blot (Fig. 2b) showed that C3 deposition on CLIO NW-CR2-CR1 was decreased compared with CLIO NW, and there was almost complete absence of the cleaved α′2 fragment. While 10 mM EDTA, a universal and established inhibitor that blocks C3 opsonization of iron oxide nanoparticles without compromising their stability,^5,23^ significantly reduced the deposition of C3 on CLIO NW, it did not further reduce the already low deposition of C3 on CLIO NW-CR2-CR1 (Fig. 2a and b). Notably, the addition of 5% (by Fe content) of CLIO NW-CR2-CR1 to CLIO NW considerably decreased the total C3 deposition (Fig. 2a and b), suggesting that CLIO NW-CR2-CR1 exhibits both *cis*- and *trans*-inhibitory activity. Since the concentration of CR2-CR1 on CLIO NW-CR2-CR1 is known (SI), the inhibitory concentration (IC_50_) was measured. CLIO NW-CR2-CR1 (up to 20% Fe) were spiked at various concentrations into CLIO NW and C3 deposition was plotted *versus* concentration. Complete inhibition was not achieved, and the IC_50_ was approximately 73 nM (Fig. 2c). While this value is lower than a single nanomolar for the soluble form of CR2-CR1,^23^ more importantly the data clearly demonstrate the ability of CLIO NW-CR2-CR1 to inhibit C3 activation on unprotected companion nanoparticles in *trans*.

**Fig. 2:**
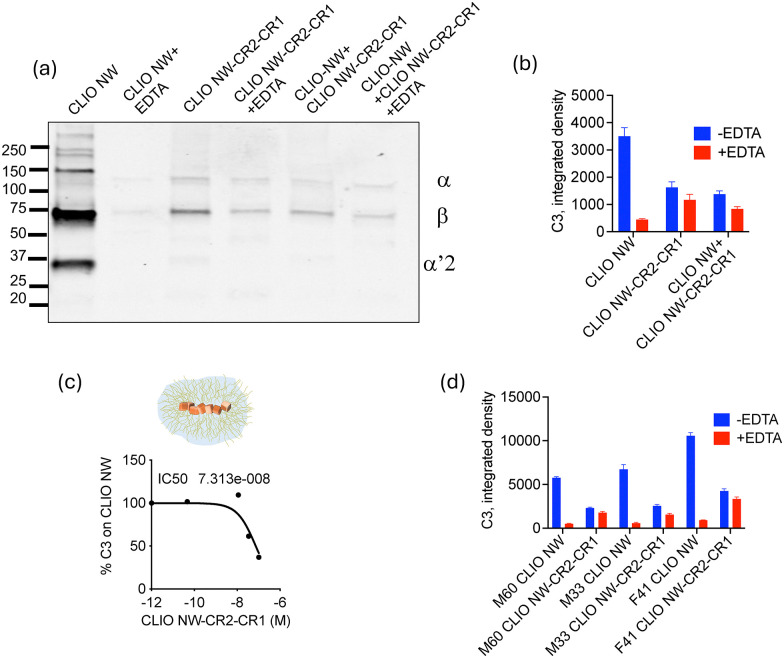
CR2-CR1 tethering to CLIO NW suppresses C3 opsonisation. Western blot (a), dot-blot (b) and (d) and IC_50_ (c) analysis of C3 opsonisation on CLIO NW-CR2-CR1 in human sera. CR2-CR1 and EDTA decreases total C3 opsonisation (decrease in β chain intensity) on CLIO NW (a)–(d) as well as inhibition of enzymatic cleavage of α chain (disappearance of α′2 fragment) (b). In (a)–(c) serum was from a 62 years old female donor. In (d) gender (M or F) and age (number) of serum donors are indicated.

Given the substantial interindividual variability in C3 deposition on nanomedicines,^32^ we tested the inhibitory effect of CLIO NW-CR2-CR1 in sera from three additional human donors. According to Fig. 2d, CLIO NW-CR2-CR1 was active in all 3 donors and was able to suppress C3 deposition and cleavage. Notably, in all donors, EDTA almost completely blocked the C3 deposition on CLIO NW, but was unable to reduce further already low C3 deposition on CLIO NW-CR2-CR1, suggesting that the residual C3 is either native C3 (non-specific deposition) or C3(H_2_O). The latter being the hydrolytic and conformationally rearranged form of C3.^1,3^

Next, we combined regulatory-approved PEGylated liposomal doxorubicin therapeutics with CLIO NW-CR2-CR1 and followed C3 opsonisation in human serum. Liposomes alone became C3 opsonised, which was in accordance with previous studies,^23^ and opsonisation was inhibited by EDTA (Fig. 3). Addition of CLIO NW-CR2-CR1 at 5% v/v (final concentration) considerably reduced C3 opsonisation of liposomes (Fig. 3). As a positive control for C3 opsonisation, CLIO NWs at 5% v/v did not affect liposome opsonisation (Fig. 3a).

**Fig. 3:**
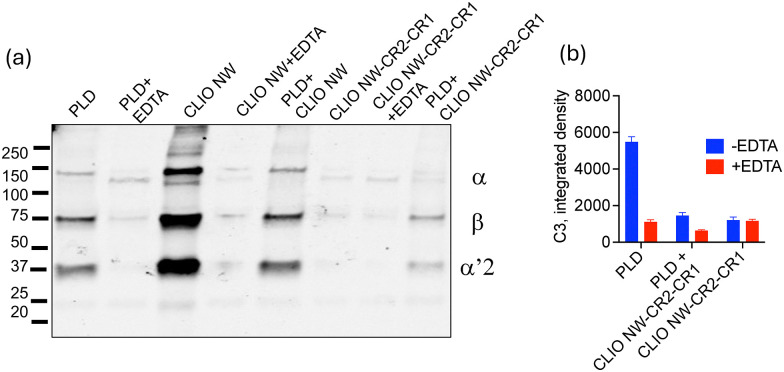
CLIO NW-CR2-CR1 suppresses C3 opsonisation of PEGylated liposomal doxorubicin (PLD). Western blot (a) and dot blot (b) analysis of inhibitory effect of CLIO NW-CR2-CR1 (5% v/v) on total C3 binding and opsonisation of PLD in a human serum (F62).

Since modification of soluble CR2-CR1 with small molecules such as different fluorophore types does not affect its complement inhibition potency,^23^ we asked whether PEG_3400_ linker might have introduced steric constraints on CR2-CR1 to binding C3 frgaments and C3 convertases. To test this, we stoichiometrically conjugated CR2-CR1 to human serum albumin (HSA) *via* PEG_3400_ spacer (SI) and tested its potency on C3 opsonisation of CLIO NWs. As shown in the SI, complete inhibition was not achieved, but the IC50 of soluble HSA-CR2-CR1 was two-fold lower (32 nM) than CLIO NW-CR2-CR1. CLIO NW-CR2-CR1 may be considered multivalent, since on average each NW is bound to 36 CR2-CR1 molecules. Therefore, it is plausible that stochastic engagement between surface-bound CR2-CR1 molecules and C3 convertases might sterically render adjacent CR2-CR1 molecules redundant. Indeed, C3 covertases are large assemblies. For instance, the alternative pathway convertase C3bBb has an asymmetrical, elongated architecture of approximately 15 nm × 10 nm × 8 nm) prior to properdin stabilisation.^33^ Furthermore, compared with soluble CR2-CR1, surface-bound CR2-CR1 might exhibit different affinities for surface-formed *versus* fluid-phase convertases, which could further impact and modulate its IC_50_. Finally, due to their cationic characteristics CR2-CR1-bound NWs could electrostatically attract anionic blood proteins. Adsorbed proteins further contribute to spatial crowding and could sterically compromise the regulatory potency of a fraction of grafted CR2-CR1 molecules.

In summary, CR2-CR1 functionalisation of nanoparticles has opened an exciting universal approach for the design and engineering of complement-evading entities, particularly with the view that such constructs inhibit C3 activation on unprotected nanoparticles in *trans* as well as PEGylated species prone to complement attack. These developments are important since derailing and overactivation of complement by nanopharmaceuticals might instigate inflammatory reactions, induce cardiovascular distress and further contribute to disease progression.^3^ For instance, intratumoural complement activation by extravasated nanoparticles was shown to promote tumour growth in an immunocompetent murine model.^34^ Considering that intratumoural complement activation promotes tumour growth through multifaceted mechanisms,^35,36^ CR2-CR1 conjugated nanoparticles might even prove useful as effective adjuvants in cancer treatment. However, further work must investigate the pharmacokinetic, biodistribution and safety profile of CR2-CR1 conjugated nanoparticles and their impact on the biological performance of nanopharmaceuticals (such as PLD), if given in combination. Finally, considering the lower potency of conjugated CR2-CR1 than its soluble form, artificial intelligence and machine learning initiatives could introduce alternative conjugation approaches that might overcome spatial crowding and orientation of the inhibitor on nanosurfaces. This could further identify approaches to new design of regulatory complement fusion constructs and nanoparticle libraries with tuneable complement responses and adjuvant properties.^37^

## Conflicts of interest

There are no conflicts to declare.

## Supplementary Material

CC-062-D6CC00733C-s001

## Data Availability

All data supporting this article are present in the paper and/or the supplementary information (SI). Supplementary information: experimental procedures and ^1^H-NMR data for MTz-PEG_3400_-COOH are included in SI. See DOI: https://doi.org/10.1039/d6cc00733c.
