# Supplementary material for: Bioorthogonal tuning of nanosurface opsonisation via click coupling of a complement fusion protein inhibitor
Source: Chem Commun (Camb). 2026 Jun 9;62(51):12859–62. doi: 10.1039/d6cc00733c (PMC13262778; doi:10.1039/d6cc00733c)

This journal is © The Royal Society of Chemistry 2026

Supplementary Information

Bioorthogonal tuning of nanosurface opsonisation via click coupling of a complement fusion protein inhibitor

Sarah Jacques,[†a] Hanmant Gaikwad,[†a] Jemma Pillatzke,[b] Seyed Moein Moghimi,[*acd] and Dmitri Simberg[*a]

[a]Department of Pharmaceutical Sciences, The Skaggs School of Pharmacy and Pharmaceutical Sciences, University of Colorado Anschutz Medical Campus, Aurora, CO, USA.
Email: Dmitri.simberg@cuanschutz.edu
[b]School of Pharmacy and Pharmaceutical Sciences, Panoz Institute, Trinity College Dublin, Dublin 2, Ireland
[c]School of Pharmacy, Newcastle University, Newcastle upon Tyne NE1 7RU, UK
Email: seyed.moghimi@newcastle.ac.uk
[d]Translational and Clinical Research Institute, Faculty of Health and Medical Sciences, Newcastle University, Newcastle upon Tyne NE2 4HH, UK
[†]Contributed equally.

MATERIALS AND METHODS

Materials. Chemicals for CLIO NW synthesis, including iron salts and epichlorohydrin, and human serum albumin were from Sigma-Aldrich (Saint Louis, MO). Pharmaceutical-grade dextran (molecular weight 20 kDa, T-20) was from Pharmacosmos (Holbæk, Denmark). Amine-poly(ethylene glycol)$_{3400}$-valeric acid (NH2-PEG$_{3400}$-VA) was from Laysan Bio (Arab, AL, USA). Trans-cyclooctene-PEG$_4$-succinimidyl ester (TCO-PEG$_4$-NHS), methyltetrazine-cyanine 3 (MTz-Cy3), and MTz-NHS were from Click Chemistry Tools (Scottsdale, AZ). EDC (1-ethyl-3-(-3-dimethylaminopropyl) carbodiimide hydrochloride) and N-hydroxysuccinimide were from Chem-Impex International, Inc., whereas sulfosuccinimidyl acetate was from Thermo Fischer. The 2x Laemmli Sample Buffer and precast Mini-PROTEAN TGX Gels were from Bio-Rad. ZebaTM Spin Desalting Columns were from Thermo Fisher Scientific. The nitrocellulose membrane (pore size 0.45 µm) was from Bio-Rad. The MACS Midi column was from Miltenyi Biotech. The goat IgG fraction against human C3 (Cat# 55033) was obtained from MP Biomedicals (Solon, OH, USA). The anti-CR2 mouse anti-human antibody (clone 1048) was provided by the VM Holers laboratory (University of Colorado). Human CR2-CR1 was provided by Dr. Tomlinson (Medical University of South Carolina) as previously described.[1,2] All proteins were stored at -80°C with less than 3 freeze-thaw cycles per aliquot.

Whole blood from healthy consented human donors (3–5mL) was collected in Vacutainer® Z tubes (without additives) at the University of Colorado Blood Donor Center, following approval of the Colorado Multiple Institutional Review Board (COMIRB) for review of human subjects research for CU Anschutz, CU Denver and affiliates, and in accordance with COMIRB guideline and protocol for anonymous sampling. The only information provided to the investigators included the donors' age and gender. Serum was obtained by separating clotted blood while adhering to strict precautions to preserve functional complement.[3,4] Serum aliquots were frozen and stored at -80 °C and were subjected to no more than two freeze-thaw cycles. PEGylated liposomal doxorubicin was obtained from the University of Colorado Cancer Center infusion pharmacy as leftover sterile vials.

Cross-linked dextran iron oxide nanocrystal preparation. Cross-linked dextran iron oxide nanocrystals were prepared as described[5] (10 mg Fe mL^{-1} in deionized distilled water) and combined with epichlorohydrin and sodium hydroxide (10N) at a 1:1:1 volume ratio. The mixture was agitated for 24 hours at 37°C and subsequently stirred with ammonium (final concentration of 2.5%) overnight at 4°C to generate cross-linked dextran iron oxide nanoworm-NH_2. The particles underwent ultrafiltration against deionized distilled water using a reverse osmosis apparatus (Pall Corporation), filtered through a 0.2µm membrane disk filter (Millipore), and stored at 4 °C.

MTz-PEG_{3400}-COOH. A combination of NH2-PEG_{3400}-VA (50 mg, 0.015 mmol, 1 equivalent), methyl tetrazine NHS (7.21 mg, 0.022mmol, 1.5 equivalents), and DIEA (N, N-diisopropylethylamine) (8 µL, 0.044 mmol, 3 equivalents) was mixed in THF at room temperature for 4 hours. The solvent was then removed under reduced pressure, and the remaining dark pink residue was purified via preparative HPLC (Isolera Biotage), eluting with a 40% to 50% methanol/water mixture to obtain MTz-PEG_{3400}-COOH as a pink solid. Yield 68.3%. ^{1}H NMR (400 MHz, $CDCl_3$); δ 9.98 (bs, 1H, NH), 8.51 (d, *J*= 8.3 Hz, 2H, Ar-H), 7.48 (d, *J*= 8.3 Hz, 2H, Ar-H), 3.38-3.81 (m, 330H, CH_2) 3.05 (s, 3H, CH_3), 2.31 (t, *J*= 7.2 Hz, 2H, CH_2), 1.53-1.72 (m, 4H, CH_2).

CLIO NW preparation. MTz-PEG_{3400}-COOH (1.44mg, 2,000 fold molar excess), EDC (0.24mg, 6,000 fold excess), NHS (0.15mg, 6,000 fold excess) were mixed in phosphate buffered saline (PBS), pH 7.4, for 30 min, then cross-linked iron oxide nanoworm-NH_2 (2mg Fe) was added to the mixture to prepare CLIO NW. The reaction mixture was stirred at 4°C for 12 hours, then a 10,000-fold excess of sulfosuccinimidyl acetate (0.5mg) was added, and the mixture was stirred for an additional 2 hours at 4°C. The reaction mixture was purified using a 40 kDa cut-off Zeba spin column.

CD2-CR1 conjugation with TCO-PEG_4-NHS. Human CR2-CR1 in PBS (200 µL, 10 mg mL^{-1}) was combined with a 10-fold excess of TCO-PEG_4-NHS (in 4 µL DMSO). The reaction mixture was incubated at 4°C for 12 h and purified using a 7 kDa cut-off Zebra spin column. Conjugation efficiency was approximately 2 TCO/CR2-CR1 molecule as determined by UV absorbance after conjugation with Cy3-MTz and the dye extinction coefficient of 1,50,000 M^{-1} cm^{-1}.

CR2-CR1-TCO conjugation with CLIO NW. CR2-CR1-TCO in PBS (100 µL, 5mg mL^{-1}) was combined with CLIO NW (100 µL, 10mg Fe mL^{-1}) at a 100-fold excess. The reaction mixture was incubated at 4°C for 12 hours and then purified using a MACS Midi column, followed by concentration using 100 kDa Amicon Ultra Centrifugal Filters. To determine the number of CR2-CR1 per nanoparticle, nanoparticles were blotted in 2 µL triplicate on a 0.45 µm nitrocellulose membrane, the membrane was blocked in 5% milk/0.1% Tween-20/PBS, and the CR2-CR1 was detected with anti-CR2 antibody and the secondary IRDye 800CW labeled antibody. The membrane was scanned at 800nm using Li-COR Odyssey. The number of CR2-CR1/NW was calculated using a standard curve applied to the same membrane. The concentration of nanoparticles used for calculations was 6×10^{13} mg^{-1} Fe.[6] The number of CR2-CR1 per NW was 36.

Preparation of HSA-PEG$_{3400}$-MTz. MTz-PEG$_{3400}$-COOH (0.53 mg, 5-fold excess), EDC (0.087 mg, 15-fold excess), NHS (0.053 mg, 15-fold excess) were mixed in PBS for 30 min, then HSA (2.0 mg) in PBS was added to the mixture. The reaction mixture was stirred at 4 ^{o}C for 12 h. The conjugation mixture was purified using a 30 kDa cut-off Amicon spin column and resuspended in PBS.

Conjugation of HAS-PEG$_{3400}$-MTz with CR2-CR1-TCO. HSA conjugated with PEG$_{3400}$-MTZ in PBS, 1 mg in PBS, and 1.5-fold excess was combined with the CR2-CR1-TCO (in PBS). The reaction mixture was incubated at 4°C for 12 h, and excess albumin was removed by purification using 100 kDa cut-off Amicon Ultra Centrifugal Filters.

Immunological assays. Quantification of C3 deposition on nanoworms was performed using a dot-blot and a Western blot. Briefly, NWs (10 µL, 1mg mL^{-1} Fe) were incubated with 30 µL human sera at 0.25 mg mL^{-1} for 30 min at 37 °C, pelleting by ultracentrifuge at 400,000g for 5 min and washed three times in PBS.[7,8] NWs were dotted on a nitrocellulose membrane for the immunodot blot assay (0.5 µg/Fe per dot) or loaded after boiling in reducing sample buffer on a SDS-PAGE gel for Western blot (5 µg Fe per lane) exactly as described previously.[7,8] Integrated dot density of dot-blot was quantified with Fiji and plotted with Prism 10 (GraphPad).

References

1. C. Atkinson, H. Song, B. Lu, F. Qiao, T. A. Burns, V. M. Holers, G. C. Tsokos, and S. Tomlinson, *J. Clin. Invest.,* 2005, **115,** 2444–2453.

2. M. Fridkis-Hareli, M. Storek, E. Or, R. Altman, S. Katti, F. Sun, T. Peng, J. Hunter, K.Johnson, Y. Wang, A. S. Lundberg, G. Mehta and N. K. Banda and V. M. Holers, *Mol. Immunol.,* 2019, **105,** 150–164

3. P. J. Lachmann, *J. Immunol. Method.,* 2010, **352,** 195–197.

4. L. P. Wu, M. Ficker, J. B. Christensen, D. Simberg, P. N. Trohopoulos and S. M. Moghimi, *Nat. Commun.,* 2021, **12,** 4858.

5. H. Gaikwad, Y. Li, G. Gifford, E. Groman, N. K. Banda, L. Saba, R. Scheinman, G. Wang, and D. Simberg, *Bioconjug. Chem.,* 2020, **31,** 1844–1856.

6. H. Benasutti, G. Wang, V. P. Vu, R. Scheinman, E. Groman, L. Saba, and D. Simberg, *Bioconjug. Chem.,* 2017, **28,** 2747–2755.

7. Y. Li, A. Monte, L. Dylla, S. M. Moghimi and D. Simberg, *J. Immunol. Method.,* 2024, **528,** 113668.

8. Y. Li, G. Wang, L. Griffin, N. K. Banda, L. Saba, E. V. Groman, R. Scheinman, S. M. Moghimi and D. Simberg, D., *J. Control. Release* 2021, **338,** 548–556.

Raw Data

Original Western Blots and Dot-Blots for Figures 2 & 3:

Fig. 2a

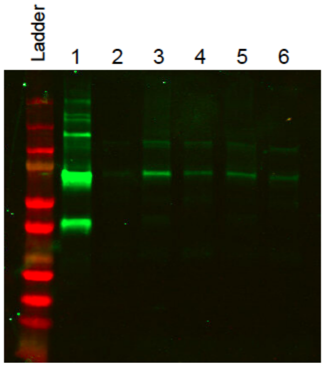

1 = CLIO NW
2 = CLIO NW + EDTA
3 = CLIO NW-CR2-CR1
4 = CLIO NW-CR2-CR1 + EDTA
5 = CLIO NW + CLIO NW-CR2-CR1
6 = CLIO NW + CLIO NW-CR2-CR1 + EDTA

Fig. 2b

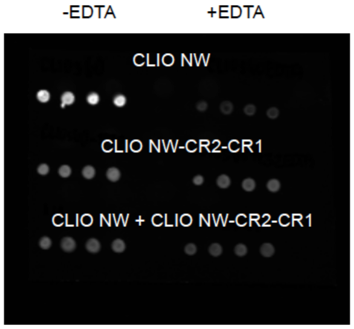

Fig. 2d

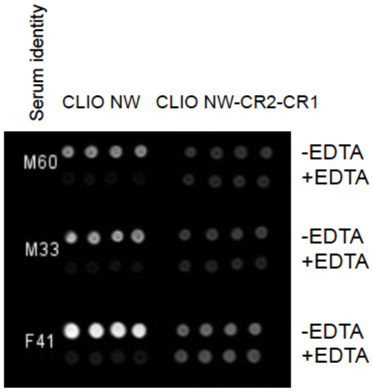

Fig. 3a

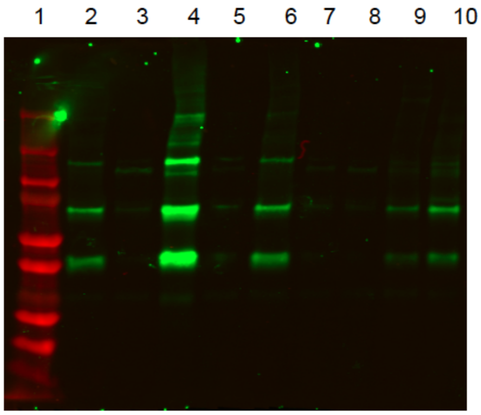

1 = Ladder
2 = PLD
3 = PLD + EDTA
4 = CLIO NW
5 = CLIO NW + EDTA
6 = PLD + CLIO NW
7 = CLIO NW-CR2-CR1
8 = CLIO NW-CR2-CR1 + EDTA
9 = PLD + CLIO NW-CR2-CR1
10 = Repeat 9

Fig. 3b

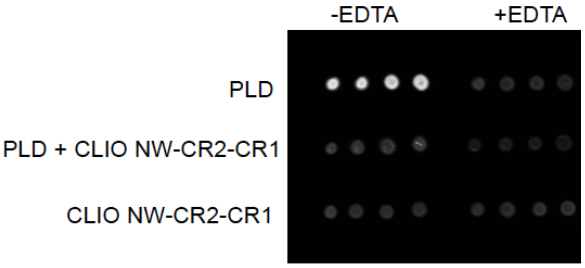

Standard curve for quantification of CR2-CR1:

CR2-CR1 mg/mL	Intensity
0.1	232.2348
0.05	136.3123
0.025	73.369
0.0125	13.2655
0.00625	6.75725
0.003125	3.74525

CLIO CR2-CR1 Intensity	CR2-CR1 on CLIO NW, mg/mL	number of CR2-CR1 per ml
1099.441	0.45	2.3E+15
		NWs per mL (at 1mg/mL Fe)
		6E+13
		CR2-CR1 per NW
		37.53020459

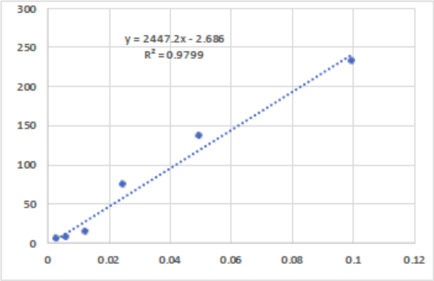

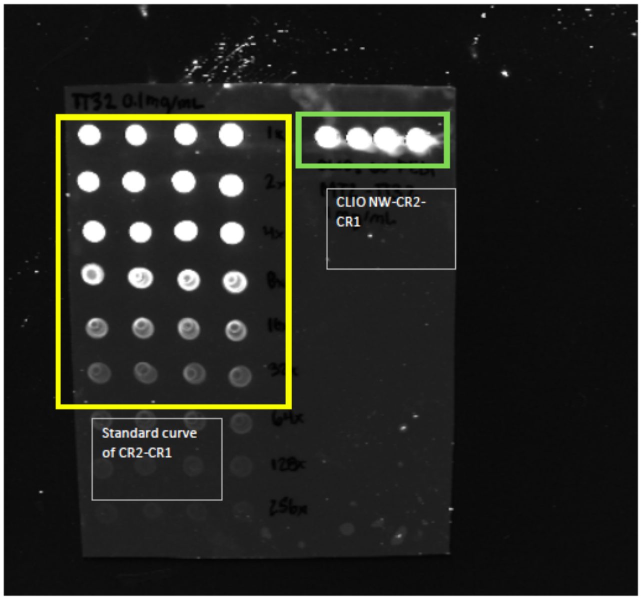

Procedures: A standard curve of CR2-CR1 was made in 1% (w/v) BSA in PBS, starting at 0.1 mg/mL and 2x, 4x, 8x, 16x, 32x dilutions thereafter. Each sample was blotted four times with 2 µL on to nicrocellulose membrane. CLIO NW-PEG-CR2-CR1 1 mg/mL was blotted four times with 2 µL onto the membrane. The membrane was dried for 10 min, blocked for 30 min in 5% (v/v) milk and probed with anti-mouse CR2 monoclonal antibody clone HB5 1:1000 overnight at 4 °C. The membrane was washed three times for 3 min, probed with anti-mouse IRDye800 (Li-COR) 1:10000 for 1h and washed three times for 3 min. The signal was detected with Li-COR Odyssey and the mean dot intensity was determined with FIJI. The concentration of CD2-CR1 in the NW dots was determined from the standard curve and for the determination of the average number of bound CR2-CR1 per NW, the number of CR2-CR1 molecules (Mw=120,000 Da) was divided by the number of NWs (6×10^{13} NW/mg).

Conjugation of human serum albumin (HAS) to CR2-CR1 (a) and analysis of the inhibitory effect HSA-CR2-CR1 on C3 opsonisation of CLIO NWs:

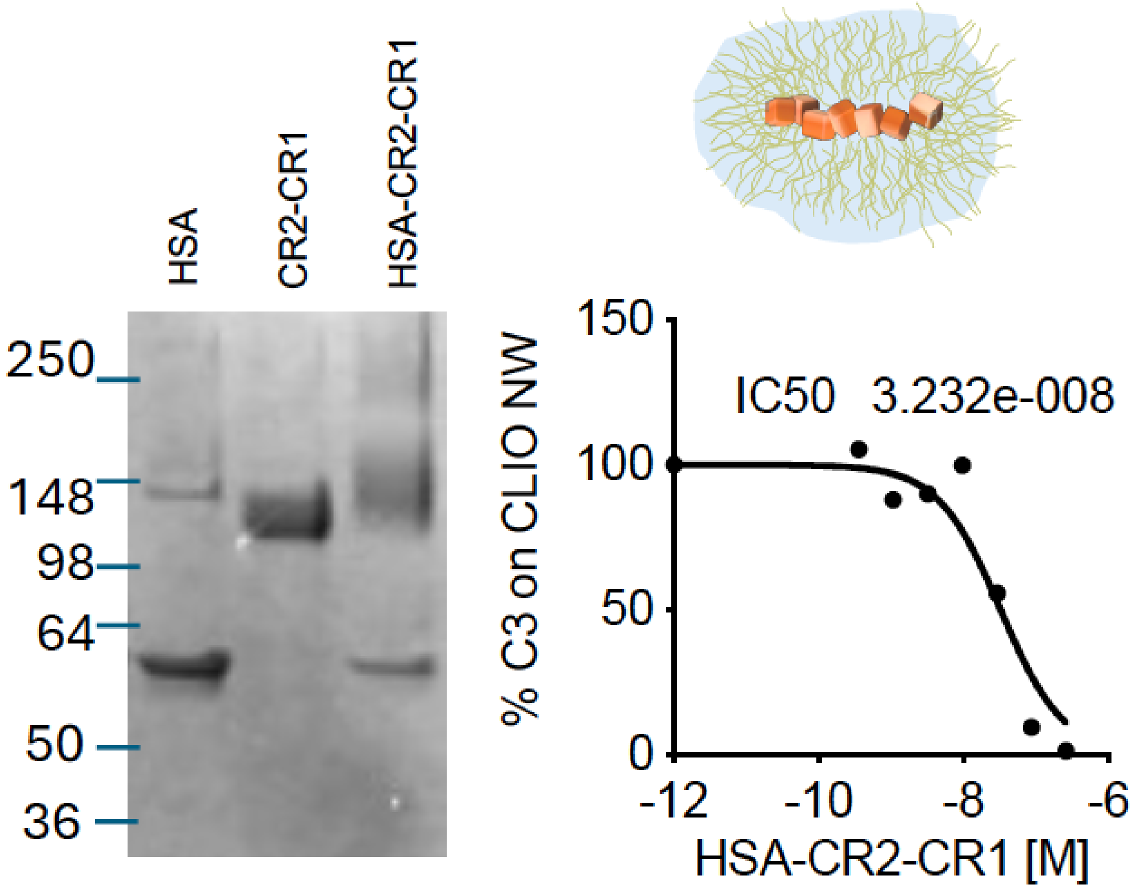

Supplement: CC-062-D6CC00733C-s001 [file CC-062-D6CC00733C-s001.pdf]
